# Therapeutic and Neuroprotective Effects of Bushen Jianpi Decoction on a Rotenone-Induced Rat Model of Parkinson's Disease

**DOI:** 10.1155/2022/9191284

**Published:** 2022-11-18

**Authors:** Wei Liang, Lulu Yao, Jiahao Chen, Zhiying Chen, Xiling Wu, Xiaobo Huang

**Affiliations:** ^1^Department of Traditional Chinese Medicine, Xuanwu Hospital, Capital Medical University, Beijing 100053, China; ^2^Department of Neurobiology, Capital Medical University, Beijing 100069, China; ^3^Department of Neurology, Xuanwu Hospital, Capital Medical University, Beijing 100053, China

## Abstract

Parkinson's disease (PD) is an age-related neurodegenerative disorder characterized by the loss of dopaminergic neurons in the substantia nigra (SN) pars compacta. Dopamine (DA) replacement therapy is one of the most effective drug treatments for PD; however, long-term levodopa treatment can lead to various side effects that negatively impact the quality of life of patients. Therefore, finding safe and effective alternative drugs to treat PD is of clinical importance. The Bushen-Jianpi decoction (BSJPD) was derived from classic traditional Chinese medicine and has been shown to be effective in the treatment of PD. This study explored the effects and mechanisms of action of BSJPD in PD. In our study, rats were randomly divided into six groups: the vehicle group, rotenone (ROT) + Saline group, ROT + low-dose BSJPD group, ROT + high-dose BSJPD group, ROT + Madopar group, and ROT + low-dose BSJPD + Madopar group. Treatment was administered to the rats once a day for 28 days, and behavioral tests were assessed. Tyrosine hydroxylase (TH), catechol-O-methyltransferase (COMT), monoamine oxidase B (MAO-B), dopa decarboxylase (DDC), alpha-synuclein (*α*-syn), and heme oxygenase-1 (HO-1) levels were detected. Our results show that BSJPD increases the body weight of rats, improves their motor coordination, reverses decreasing TH levels in the SN, and increases the expression level of DDC and HO-1 in the striatum (ST), but it fails to affect TH levels in the ST in the PD model. In addition, BSJPD reduced the expression of MAO-B in the ST in the PD model, but it did not have a significant effect on COMT. Rather, COMT in the plasma and liver increased in the low-dose BSJPD treatment group. Upregulation of *α*-syn in the PD model was also observed, but BSJPD has shown no obvious effect to clear it. Our results suggest that BSJPD exhibits a therapeutic effect on PD and may play a neuroprotective role by regulating HO-1 expression and participating in the metabolic process of DA.

## 1. Introduction

Parkinson's disease (PD) is a progressive neurodegenerative movement disorder, with an incidence of approximately 1% in the population over 60 years of age [1]. In PD, motor symptoms such as bradykinesia, rigidity, resting tremor, and postural instability occur due to the loss of nigrostriatal dopamine (DA) neurons [2], which are vital for the proper functioning of the basal ganglia pathway. L-DOPA (levodopa), the precursor of DA, is the most effective drug for treating PD. Currently, Madopar® (L-dopa + benserazide) is the gold standard treatment for PD, but long-term use of this drug may result in a variety of serious adverse effects, leading to fluctuations in motor symptoms such as L-dopa-induced dyskinesia (LID), which limits its therapeutic effect in PD and diminishes the quality of life of patients with PD. Moreover, Madopar® is not very effective against nonmotor PD symptoms such as anxiety, depression, sleep disorders, pain, and constipation. Owing to the diversity of PD symptoms, a battery of nondopaminergic therapies has been administered in PD but has yielded limited clinical benefits. Strategies employing potential synergistic actions in combination with Madopar® may provide novel alternative therapies for PD.

Bushen-Jianpi decoction (BSJPD) is a traditional Chinese herbal compound that can invigorate the spleen and tonify the kidney. Its main components are presented in Table 1. The Gegen decoction, one of the earliest Chinese herbal compounds, has been used as a prescription for back pain, muscle rigidity, and vertigo [3]. Puerarin is one of the most important active components of *Pueraria lobate* (Willd.) Ohwi and has been shown to possess anti-inflammatory [4], antioxidant [5], vascular expansion [6], and analgesic effects. It is widely used in the treatment of neurodegenerative diseases [7] such as pain, cerebrovascular diseases [8], Alzheimer's disease [9], and PD [5, 10]. The Zhizhu decoction was prescribed for the treatment of gastrointestinal indigestion in ancient China and can promote gastrointestinal movement and reduce symptoms of discomfort, such as abdominal distension and abdominal pain [11]. *Rehmannia glutinosa* Libosch is a traditional kidney-tonifying Chinese herb that can reduce neuronal apoptosis, clear *α*-synuclein (*α*-syn), and inhibit MAO-B activity, thereby protecting neurons. *β*-asarone is one of the active components of *Acorus tatarinowii* Schott, which can effectively decrease *α*-syn expression, increase DA and L-dopa contents of the striatum (ST), and increase MAO-B and COMT levels [12, 13]. Apoptosis and autophagy play a vital role in PD pathogenesis, and plenty of herbs have been shown to effectively restore the balance between apoptosis and autophagy [14–17].

One of the models of PD that has been developed is the ROT-induced PD model. The insecticide rotenone (ROT) is widely used in agricultural production to selectively inhibit the activity of mitochondrial complex I, which can cause mitochondrial dysfunction and insufficient energy supply. ROT can produce a large amount of reactive oxygen species (ROS) that damage the nigrostriatal dopaminergic system, which is highly sensitive to oxidative stress. In 2000, Betarbet et al. was the first to demonstrate that ROT can cause selective damage to the nigrostriatal dopaminergic system in rats as well as the aggregation of *α*-syn in DA neurons. The authors reproduced the pathological characteristics [18] of PD in a rat model. The ROT model was previously used to investigate the pathogenesis of PD and assess the effectiveness of neuroprotective drugs. Importantly, age plays an important role in the onset of PD and is a key factor in sporadic PD. Moreover, the loss of DA neurons in the substantia nigra (SN) increases with age, leading to an increased risk of PD. It has been suggested that the use of rodents of middle-age (12–14 months) enables the development of reproducible rotenone-induced PD models and decreases model variation [19].

To the best of our knowledge, no previous study has investigated the effects of BSJPD on PD. Therefore, in this study, we explored the efficacy of BSJPD in a rotenone-induced PD model using middle-aged rats.

## 2. Materials and Methods

### 2.1. Animals

Fifty-one middle-aged male Wistar rats (age: 9–10 months, weight: 550–650 g) were obtained from Beijing Vital River Laboratory Animal Technology Co., Ltd. (Beijing, China). The experimental protocol was approved by the Institutional Animal Care and Use Committee of Xuanwu Hospital (permit no. XW-20210902-1). All rats were reared under standardized housing conditions (12/12 h light/dark cycle, temperature, 23 ± 2°C, and relative humidity, 50% ± 5%) and were provided with *ad libitum* access to food and water.

### 2.2. Chemicals and Reagents

ROT was purchased from Sigma-Aldrich (R8875, St. Louis, MO, USA), Madopar® was purchased from Roche Pharmaceutical Co., Ltd. (Shanghai, China), and sunflower oil was purchased from Shanghai Yuanye Biotechnology Co., Ltd. (S24927, Shanghai, China). Rabbit anti-tyrosine hydroxylase (TH) primary antibody, mouse anti-alpha-synuclein (*α*-syn) antibody, rabbit anti-catechol-O-methyltransferase (COMT) antibody, rabbit anti-monoamine oxidase B (MAO-B) antibody, rabbit anti-dopa decarboxylase (DDC) antibody, and rabbit anti-heme oxygenase-1 (HO-1) antibody were all purchased from Abcam (ab137869, ab280377, ab126618, ab259928, ab131282, and ab68477, respectively; Cambridge, UK). Mouse anti-*β*-actin antibody was purchased from Huaxing Bio Specialist in Biological Medicine (HX1827; Beijing, China), rabbit anti-nuclear factor erythroid 2-related factor 2 (Nrf2) antibody was purchased from Immunoway (YT3189; Plano, TX, USA), and secondary antibodies linked to fluorescein (FITC)-conjugated AffiniPure Donkey Anti-Rabbit IgG (*H* + *L*) were purchased from Proteintech (SA00003-8, Wuhan, China).

### 2.3. Herbal Extract

The herbal composition of BSJPD is shown in Table 1. All herbs were purchased from Xuanwu Hospital, Capital Medical University (Beijing, China), soaked in 2 L of water for 1 h, boiled for 30 min, filtered for three times, and concentrated for 1 h. Finally, an aqueous extract concentration of 2.3 g/mL of aqueous extract was obtained.

### 2.4. Experimental Design

**Figure 1:**
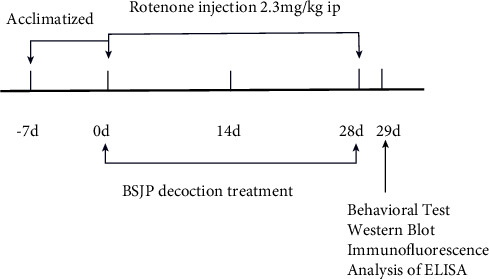
Flow chart illustrating the experimental design.

The rats were acclimatized for 1 week before the experimental procedure. They were then randomly divided into six groups: the vehicle group (*n* = 8), injected with sunflower oil, ROT + Sal group (*n* = 9), injected with ROT and treated with 0.9% saline, ROT + *L* group (*n* = 8), injected with ROT and treated with low-dose BSJPD (7 g/kg body weight), ROT + *H* group (*n* = 8), injected with ROT and treated with high-dose BSJPD (14 g/kg body weight), ROT + Madopar® (*n* = 9), injected with ROT and treated with Madopar® (50 mg/kg body weight), and ROT + *L* + Madopar®(*n* = 9), injected with ROT and treated with a low dose of BSJPD plus Madopar® (50 mg/kg). We prepared the ROT solution (2.3 mg/mL of ROT in 98% sunflower oil, 2% DMSO, V/V) [20], and the dosage of ROT used was based on our pilot study (data not shown). ROT (2.3 mg/kg body weight) was administered intraperitoneally once a day for 28 days. Vortexing of the solution created a stable suspension of DMSO containing ROT and sunflower oil. A fresh solution was prepared twice a week, stored in plastic bottles wrapped with tinfoil to protect it from light, and inverted several times before each round of injections to eliminate the possibility of settling. The experimental flow is illustrated in Figure 1. The rats were weighed in the morning, BSJPD was administered intragastrically, and ROT was administered 4 h later once per day for 28 days.

### 2.5. Behavioral Tests

#### 2.5.1. Open-Field Test

The rats were allowed to freely explore an open-field box (1 m × 1 m × 40 cm) for 5 min. The time spent in the central area and the total distance travelled were videotaped and quantified with EthoVision for each 5 min period (Noldus, VA, USA). The field was cleaned with 75% ethanol between tests [21].

#### 2.5.2. Rotarod Test

The rotarod test was used to evaluate motor coordination and balance in rats. Rats were placed on a slowly accelerating rod so that they would try to maintain balance and avoid falling. Before initiating the experiment, the rats were trained for three consecutive days to adapt to the rotating rod (three sessions per day, 5 min each). We then recorded the time taken for the rats to walk on the rotating rotarod. The maximum time for a rat to walk on the rotarod was 5 min [22].

### 2.6. Western Blot Analysis

After the rats were decapitated, their brains were quickly removed and placed on ice. SN and ST tissues were quickly isolated and frozen in dry ice for 30 min, and samples were transferred to a −80°C refrigerator for subsequent enzyme-linked immunosorbent assay (ELISA) and western blot detection. The frozen SN and ST tissues were then homogenized on ice in RIPA buffer (Applygen, Beijing, China) and centrifuged at 12,000 rpm at 4°C for 20 min to isolate the supernatant. The protein concentration was determined using a BCA protein assay kit (P0012, Beyotime, Beijing, China). The samples were run on 10% SDS-PAGE gels, with a total volume of 20–30 *μ*g of protein loaded per lane. The separated proteins were transferred on a PVDF membrane using a semidry transfer system (Bio-Rad, Hercules, CA, USA). After blocking with 5% nonfat milk in TBST for 2 h at room temperature or NumBlot blocking buffer (New Cell and Molecular Biotech Co., Ltd, Jiangsu, China) for 20 min at room temperature, the membranes were incubated overnight at 4°C with the following primary antibodies: TH (Abcam, 1 : 1000), MAO-B (Abcam, 1 : 2000), COMT (Abcam, 1 : 1500), DDC (Abcam, 1 : 1000), Nrf2 (Immunoway, 1 : 1000), HO-1 (Abcam, 1 : 1000), and *α*-synuclein (Abcam, 1 : 1000). They were then washed with TBST and incubated with either anti-rabbit (1 : 10000) or anti-mouse secondary antibodies (Proteintech, 1 : 10000) for 1 h at room temperature. After washing three times, the bands on the membrane were scanned using an Odyssey® Infrared Imaging system (LI-COR, NE, USA). *β*-actin was used as an internal standard to monitor loading errors. ImageJ analysis software (NIH, MD, USA) was used to quantify the signals.

### 2.7. Immunofluorescence

Rats were deeply anesthetized by intraperitoneal injection of 2% pentobarbital sodium, and the chest was quickly dissected to expose the heart. Blood samples were collected from the aortic artery of each rat. The needle from the left apex was subsequently infused through the left cardiac aorta with approximately 200 mL of 0.01 M PBS at 37°C (containing heparin sodium, 0.14 g/L), followed by 300–400 mL of precooled 4% paraformaldehyde (PFA,w/v). The head of each rat was dissected; the entire brain was removed, fixed with 4% PFA (w/v, 4°C for 24 h), and successively dehydrated using 20% (w/v) and 30% (w/v) sucrose solutions. Brain tissue was considered to be sufficiently dehydrated once it completely sank to the bottom of the 30% sucrose solution. Subsequently, the brain was wrapped in tin foil, flash frozen in dry ice for 20–30 min, and stored at −80°C. The brains of the rats were cut coronally (30 *μ*m) with a freezing microtome (LEICA CM3050S, Wetzlar, Germany) and stored in cryoprotectant at −20°C until use. Then, 0.4% PBST was used to break the membranes of the rat brain sections at room temperature for 30 min, and they were blocked for 1 h in 0.4% PBST containing 3% fetal bovine serum. The sections were then washed three times and incubated with rabbit anti-TH antibody (1 : 1000, Abcam) overnight at 4°C. The brain sections were then washed with 0.01 M PBS three times and further incubated in a corresponding fluorescence-conjugated donkey anti-rabbit secondary antibody (1 : 300, Proteintech, Wuhan, China) for 1 h at room temperature. After washing three times with 0.01 M PBS, the brain slices were sealed with an antifade mounting medium containing DAPI [23]. Finally, immunostaining images were acquired using the Panoramic SCAN II system (3DHistech, Budapest, Hungary).

### 2.8. TH-Positive Cell Counting in the SN

The rat brain was cut into 30 *μ*m sections using a freezing microtome. A total of 48 consecutive brain slices were collected from the SN. One slice was taken for every sixth interval, and a total of eight slices were taken from each specimen brain for immunofluorescence analysis [24] (five to eight slides from each rat were analyzed). TH-positive cells were counted in a blinded fashion using ImageJ software (NIH, MD, USA). All images were converted into binary mask images, followed by setting the shape and size of the cell. All particles of a fixed size were counted using the software. Finally, the mean values of the TH-positive cell numbers were calculated by averaging the number of brain slices for each rat.

### 2.9. Analysis of MAO-B and COMT Using ELISA

#### 2.9.1. Plasma

The whole blood of rats was collected in tubes containing EDTA, and the blood was centrifuged (TOMY MX-307, Tokyo, Japan) for 20 min at 3000 rpm at 4°C within 30 min of collection. We then collected the supernatant and stored it at −80°C for later use.

#### 2.9.2. Tissue Homogenates

The liver and striatum tissues were rinsed with 1 × PBS (pH 7.4) to remove excess blood, homogenized in 1 × PBS (pH 7.4), and stored overnight at −80°C. After two freeze-thaw cycles were performed to break the cell membranes, the homogenates were centrifuged for 15 min at 3000 rpm. The supernatant was collected immediately and stored at −80°C.

The levels of MAO-B and COMT in the plasma, liver, and ST were determined using ELISA kits on a microplate reader (Bio-Rad, CA, USA) according to standard protocols (ml037160, ml037128, Shanghai Enzyme-linked Biotechnology Co., Ltd., China). A 20 *μ*L plasma sample or tissue homogenate was diluted at a ratio of 1 : 5 in a diluent, and 100 *μ*L of streptavidin-HRP was added; the plates were incubated at 37°C for 1 h and then washed with a buffer five times. Subsequently, 50 *μ*L of substrates A and B was added and incubated at 37°C for 15 min. The OD value was measured at 450 nm after the addition of 50 *μ*L of stop solution. A few plasma samples were discarded because of severe hemolysis.

### 2.10. Statistical Analysis

Statistical analysis was performed using GraphPad Prism version 8.0.1 (GraphPad, San Diego, CA, USA). Numerical data that were normally distributed were analyzed using a one-way analysis of variance (ANOVA) followed by Tukey's or Sidak post hoc analysis. Due to unequal variances, data were analyzed using Brown–Forsythe and Welch ANOVA tests with Tamhane's T2 post hoc analysis. Non-normally distributed data were analyzed using the Kruskal–Wallis test with Dunn's post hoc analysis. Outliers in each group were identified and removed using box plot analysis [25]. Data are presented as mean ± standard error of the mean (SEM). Statistical significance was set at *P* < 0.05.

## 3. Results

### 3.1. Changes in Body Weight and Behavior

**Figure 2:**
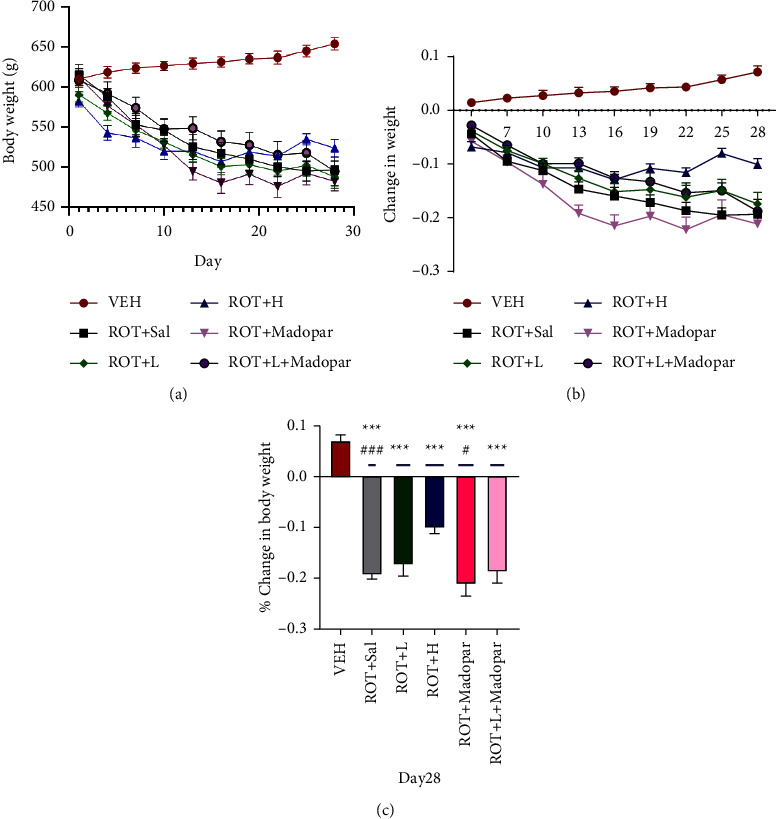
Changes in body weight during BSJPD treatment. (a) The body weight of rats, (b) change in body weight from Day 1 to Day 28, and (c) change in body weight at Day 28. Data are shown as mean ± SEM. Data were analyzed using Brown–Forsythe and Welch ANOVA tests followed by Tamhane's T2 multiple comparisons test, *n* = 7–9 per group. ^*∗∗∗*^compared with VEH group; ^#^compared with ROT + *H* group (^*∗∗∗*^*P* < 0.001, ^#^*P* < 0.05, and ^###^*P* < 0.001).

Daily intraperitoneal injections of ROT caused progressive weight loss and behavioral deficits in rats. As shown in Figures 2(a) and 2(b), the body weight of rats in the ROT + *H* group began to decrease on day 4 following ROT administration (it decreased by 6.82 ± 1.01%), gradually stabilized after 2 weeks (decreased by 12.9% ± 0.98%), and slowly reversed at the experimental endpoint (decreased by 10.09% ± 1.1%). The rats in the ROT + *H* group exhibited less weight loss than those in the ROT + Sal group (Figure 2(c), *P* < 0.001). There was no significant increase in body weight in the Madopar® group compared to that in the ROT + Sal group.

**Figure 3:**
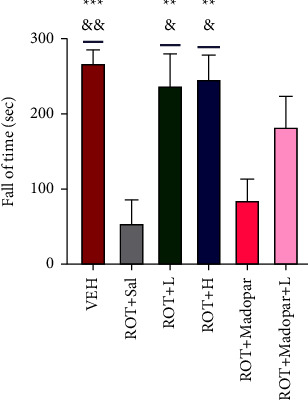
Amount of time rats spent walking on the rotarod. Data are shown as mean ± SEM. Data were analyzed using by one-way ANOVA followed by Sidak post hoc analysis, *n* = 6–8 per group. ^*∗*^compared with the ROT + Sal group and ^&^compared with the ROT + Madopar® group (^*∗∗∗*^*P* < 0.001, ^*∗∗*^*P* < 0.01, and ^&&^*P* < 0.01, ^&^*P* < 0.05).

**Figure 4:**
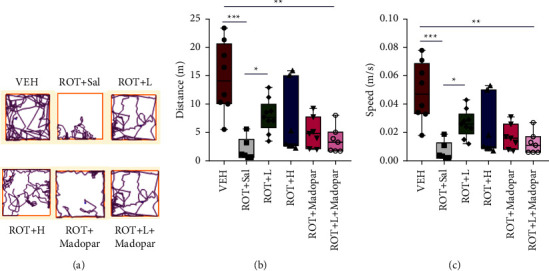
Distance travelled and speed of rats in the open-field test. (a) Track plots for open-field test, (b) total travelled distance, and (c) mean speed. Data are shown as mean ± SEM. Data were analyzed using Kruskal–Wallis test followed by Dunn's post hoc analysis, *n* = 6–9 per group (^*∗∗∗*^*P* < 0.01, ^*∗∗*^*P* < 0.01, and ^*∗*^*P* < 0.05).

Compared to the performance of rats in the ROT + Sal and ROT + Madopar® groups, the time that the rats walked on the rotarod increased in the VEH, ROT + *L*, and ROT + *H* groups (Figure 3, *P* < 0.01 and *P* < 0.05). However, no significant change was observed in the ROT + *L* + Madopar ® group. As indicated in Figures 4(a)–4(c), the total distance travelled and speed of rats in the open-field test improved in the ROT + *L* group.

### 3.2. Mortality and Variability

According to a previous study and the results of our study, acute toxic effects (PD-like symptoms) of ROT mostly appeared one week after drug administration [26], which included postural balance disorder, rigidity, and bradykinesia. In fact, these are acute nonspecific symptoms caused by ROT that do not appear as a result of PD. The rats developed dysphagia and gastrointestinal emptying disorders when they were poisoned [27], and they lost the ability to eat and drink, resulting in significant weight loss and death. Dissection of the abdominal cavity revealed swelling of the gastrointestinal tract which contained large amounts of undigested food and feces; we observed partial necrosis of the liver and poor overall health. In addition, the rats showed bleeding in the corners of their eyes, claws, and nasal mucosa. Some rats also exhibited wheezing. Three rats in the ROT + Sal group developed severe symptoms within 7 days of ROT administration. They were then provided with assisted feeding (twice a day), and the ROT injection was discontinued for 1–2 days but one still died. In addition, three rats in the ROT + Madopar® group and one in the ROT + *H* group developed similar symptoms, and one in each group died. Rats that showed early symptoms of ROT poisoning but did not die showed some resistance to ROT upon subsequent administration, including a mild gain in body weight, gradual recovery of locomotor ability, and no PD symptoms during the later stage. No animals exhibited PD-like symptoms in the VEH, ROT + *L*, and ROT + *L* + Madopar® groups in 14 days (Table 2) when ROT injection began. Therefore, nonspecific symptoms induced by ROT may be delayed or attenuated by BSJPD treatment.

### 3.3. Western Blot

**Figure 5:**
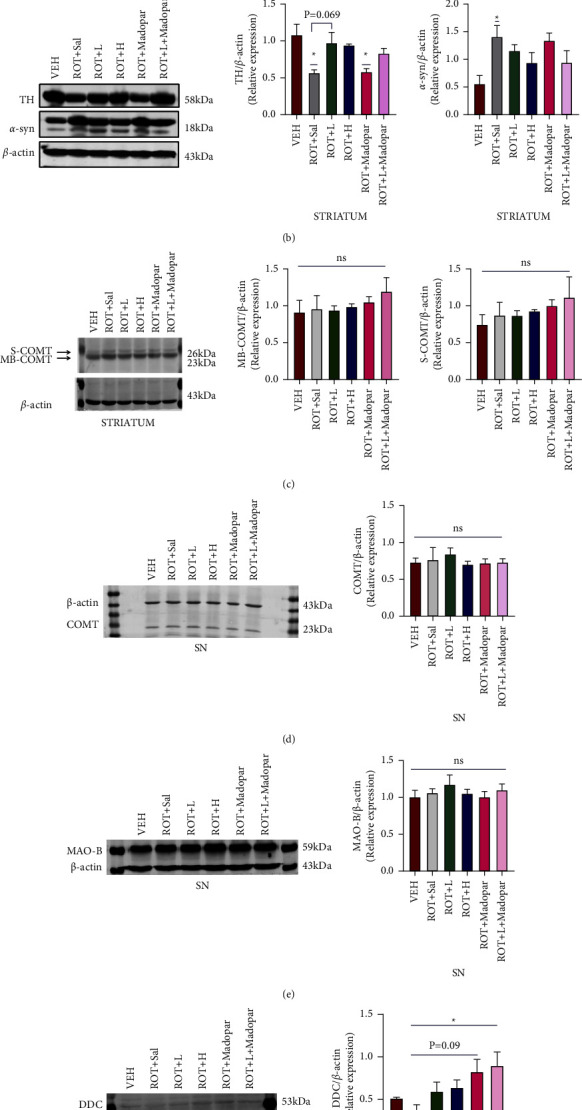
Analysis of DA-metabolizing enzymes in the SN and ST. (a) Western blot with TH and *α*-syn in the SN (*n* = 3), (b) western blot with TH and *α*-syn in the ST (*n* = 3), (c) western blot with S-COMT and MB-COMT in the ST (*n* = 3), (d) western blot with COMT in the SN (*n* = 3), (e) MAO-B in the SN (*n* = 3), and (f) DDC in the ST (*n* = 3). Data are shown as mean ± SEM. Data were analyzed using one-way ANOVA followed by Tukey post hoc analysis. ^*∗*^Compared with the VEH group, ^#^compared with the ROT + Sal group, and ^&^compared with the ROT + Madopar® group, (^*∗∗∗*^*P* < 0.001, ^*∗∗*^*P* < 0.01, ^*∗*^*P* < 0.05, ^##^*P* < 0.01, and ^&&^*P* < 0.01).

The expression of TH in the SN and ST regions was detected using western blotting. As shown in Figure 5(a), the highest expression of TH occurred in the VEH group, whereas significant reductions were observed in the other groups (^*∗∗∗*^*P* < 0.001 and ^*∗∗*^*P* < 0.01). TH expression was upregulated in the ROT + *L*, ROT + *H*, and ROT + *L* + Madopar® treatment groups (^##^*P* < 0.01). In the ST region, TH expression decreased significantly in the ROT + Sal and ROT + Madopar® groups compared with that in the VEH group (^*∗*^*P* < 0.05, Figure 5(b)). Moreover, only a moderate difference in TH levels was observed in the ROT + *L* group compared with that of the ROT + Sal group (*P*=0.069, Figure 5(b)), and no significant difference was observed in the TH levels of other groups.

*α*-syn, a pathological hallmark of PD, is a major component of Lewy bodies and is found in both familial and sporadic PD [28]. Abnormal aggregation of *α*-syn is a major trigger of neurodegenerative diseases. In animal models of PD [29, 30], ROT was reported to induce increased expression of *α*-syn, which leads to DA neuronal damage. In our study, the expression levels of *α*-syn were significantly higher in the ROT + Sal group than in the VEH group (*P* < 0.05, Figures 5(a) and 5(b)), and no significant differences were observed in the other groups. Furthermore, there was no obvious difference in the protein expression of COMT and MAO-B (Figures 5(c)–5(e)). Increased expression of DDC was observed in the ROT + *L* + Madopar® group compared to that in the ROT + Sal group (*P* < 0.05, Figure 5(f)). Nevertheless, no difference was observed between the ROT + Madopar + *L* and ROT + Madopar groups (Figure 5(f)).

**Figure 6:**
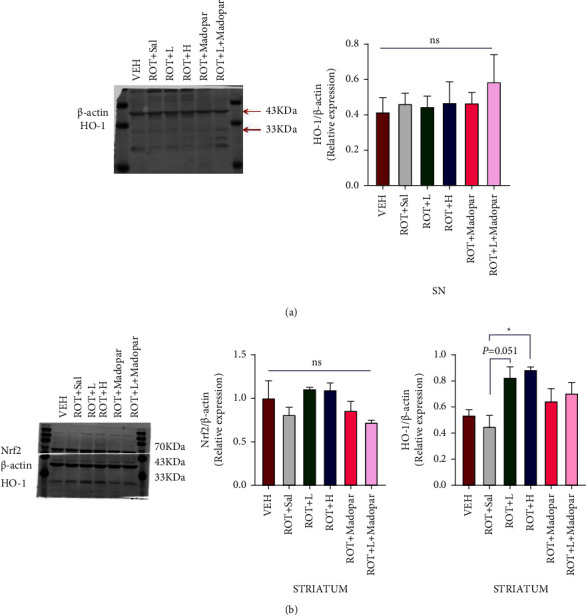
Levels of Nrf 2 and HO-1 in SN and ST. (a) Western blot with HO-1 in the SN (*n* = 3) and (b) western blot with Nrf 2 and HO-1 in the ST (*n* = 3). Data are shown as mean ± SEM. Data were analyzed using one-way ANOVA followed by Tukey post hoc analysis (^*∗*^*P* < 0.05).

Nrf2, a cytoprotective factor, can regulate the expression of genes coding for antioxidant and anti-inflammatory [31] factors. Nrf2 activation initiates the expression of phase II enzymes such as HO-1 and NADPH quinone oxidoreductase 1 (NQO1) [32]. When the body is under oxidative stress, the expression of the Nrf2/HO-1 signaling pathway increases, allowing it to effectively remove ROS and reduce cell damage. In our results, a lower expression level of Nrf2 in the SN may have occurred, which could not be detected, and no significant difference was found in HO-1 expression (*P* > 0.05, Figure 6(a)). In addition, no obvious increase was observed in the expression of Nrf2 in the ST group, while the level of HO-1 was upregulated in the ROT + *H* (*P* < 0.05, Figure 6(b)) and ROT + *L* (*P* = 0.051, Figure 6(b)) groups.

### 3.4. COMT and MAO-B Levels in the Plasma, Liver, and ST

**Figure 7:**
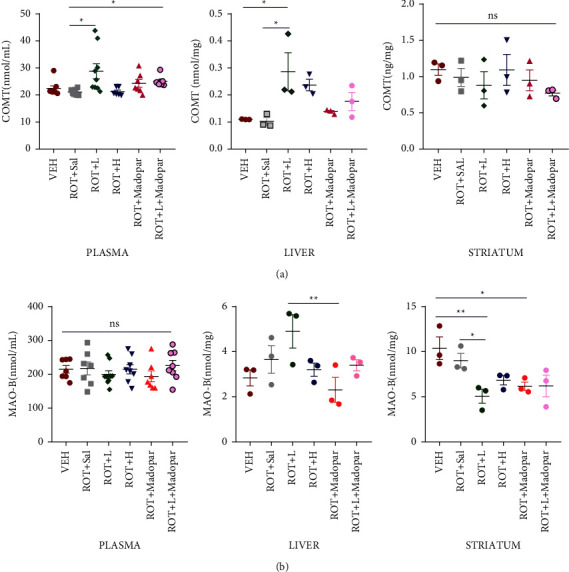
MAO-B and COMT contents in the plasma, liver, and ST. (a) COMT content in the plasma (*n* = 6–9), liver, and ST (*n* = 3) and (b) MAO-B content in the plasma (*n* = 7–9), liver, and ST (*n* = 3). Data are shown as mean ± SEM. Data were analyzed using the Kruskal–Wallis test followed by Dunn's post hoc analysis or one-way ANOVA followed by Tukey post hoc analysis (^*∗*^*P* < 0.05 and ^*∗∗*^*P* < 0.01).

Inhibition of peripheral COMT enzymes can reduce the degradation of L-dopa and increase the amount that enters the brain. Moreover, central inhibition of COMT enzymes reduces the metabolism of both L-DOPA and DA, thus increasing the half-life of both substances. Additionally, a decrease in the level of MAO-B can slow down the metabolic process of DA, cause an increased concentration of DA in the brain, and contribute to alleviating PD symptoms. We measured the levels of COMT and MAO-B enzymes in the plasma, liver, and ST to explore the effect of BSJPD on their expression levels. The COMT content significantly increased in the plasma and liver in the ROT + *L* treatment group (Figure 7(a), *P* < 0.05), but there was no significant difference in the ST (Figure 7(a)). Moreover, MAO-B levels in the ST were significantly decreased in the ROT + *L* and ROT + Madopar® groups (Figure 7(b), *P* < 0.05 and *P* < 0.01).

### 3.5. Immunofluorescent Staining of TH-Positive Neurons

**Figure 8:**
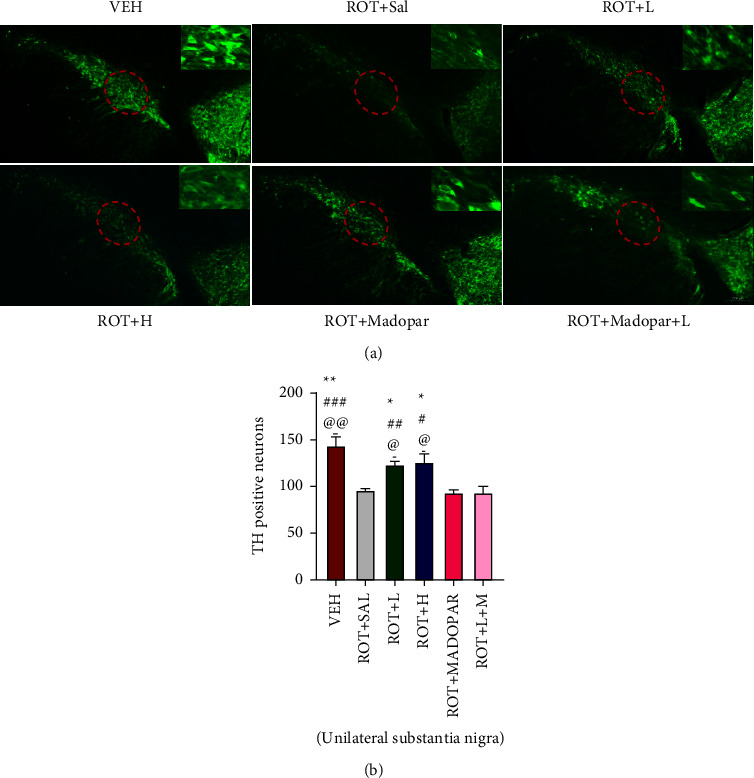
Number of TH-positive cells in the SN. (a) Representative photomicrographs of TH-positive neurons in the SN (Bar = 200um) and (b) quantification of TH-positive cells in the SN (unilateral SN). ^*∗*^Compared with the ROT + Sal group, ^#^compared with the ROT + Madopar® group, and ^@^compared with the ROT + *L* + Madopar® group. Data are shown as mean ± SEM. Data were analyzed using one-way ANOVA followed by Sidak post hoc analysis (^*∗*^*P* < 0.05, ^*∗∗*^*P* < 0.01, ^#^*P* < 0.05, ^##^*P* < 0.01, ^##^*P* < 0.001, ^@^*P* < 0.05, ^@^*P* < 0.01, and ^@@^*P* < 0.01).

TH-positive cells were scanned using immunofluorescence staining to observe the number of DA neurons. As illustrated in Figure 8, TH-positive cells showed a significant decrease in the ROT + Sal, ROT + Madopar®, and ROT + *L* + Madopar® groups. The BSJPD treatment group exhibited an increase in the number of TH-positive cells.

## 4. Discussion

Rotenone, a common pesticide, is believed to exert effects that are closely related to the onset of PD and is widely used as an environmental toxin to induce PD models. It has many advantages over other environmental toxins and can reproduce key characteristics of PD, including systemic mitochondrial damage, oxidative stress, selective nigrostriatal dopaminergic degeneration, and *α*-syn aggregation. However, because of its variability, the use of this model in PD research has been severely restricted. According to the results of some studies, ROT model rats have a high mortality rate and encounter nonspecific symptoms [18] such as severe indigestion, intestinal obstruction, liver injury, and low body weight. To decrease the variability of the ROT model and given that age plays a vital role in the pathogenesis of PD, middle-aged rats (9-10 months) were used in our protocol. It has also been reported that the ROT model only causes striatal lesions and that no significant damage occurs in the SN. In this study, we found that except for early noncharacteristic damage within two weeks, the rats developed severe PD phenotypes, such as bradykinesia, rigidity, and postural instability. However, there was still some variability in the duration from the start of ROT treatment to the onset of severe PD symptoms. Therefore, the variability of the model could not have been caused by age alone, and BSJPD may have been able to relieve nonspecific symptoms.

Open-field and rotarod tests are often used to assess locomotor ability, motor balance, and coordination in rats and have been widely used to evaluate the effects of drugs. In our study, rats in the VEH and BSJPD treatment groups moved faster, travelled longer distances, and walked for a longer period of time on the rotarod, which indicates that BSJPD could improve locomotor ability and balance coordination ability.

TH, a rate-limiting enzyme during DA synthesis, is present in the cytoplasm of DA neurons. A significant loss of TH-positive cells was observed in the ROT model group (Figure 8), whereas it was increased in the ROT + *L* and ROT + *H* treatment groups, which was consistent with the trend of the expression level of TH detected by western blot (Figure 5(a)). Therefore, BSJPD may have neuroprotective effects in the ROT-induced rat model of PD. Increased expression of *α*-syn has previously been reported in a ROT-induced PD model, and this was confirmed by immunofluorescence and western blot assays [18, 33]. Our western blot experiments also revealed that *α*-syn was highly expressed in the SN and ST areas, but BSJPD did not play a role in its removal.

DDC, COMT, and MAO-B are three major DA-metabolizing enzymes, and changes in their contents in the peripheral and central nervous system can affect the metabolic process of L-dopa [34]. Therefore, they are often used as therapeutic targets in screening for effective drugs to treat PD [35]. According to our clinical observations, BSJPD can improve the symptoms of PD and decrease the oral doses of Madopar® while extending the “ON” period of PD. Hence, we explored the mechanism action of BSJPD in PD and its influence on the levels of DDC, COMT, and MAO-B enzymes. As shown in Figure 5(f), the DDC level was significantly increased in the ROT + *L* + Madopar® group (*P* < 0.05), which indicated that ROT + *L* + Madopar® treatment may increase the expression level of DDC and may increase the percent of L-DOPA change to DA. However, no significant synergistic effect was observed between the ROT + *L* + Madopar® and the ROT + Madopar® group; however, there may have been a slightly enhanced effect. No significant changes in COMT were observed in the SN or ST (Figures 5(c) and 5(d), *P* > 0.05). However, the COMT content only increased in the ROT + *L* group in both the plasma and liver. There are two possible explanations for our results. One possible reason is that the COMT content increased owing to increased cellular apoptosis and COMT release into the peripheral blood. However, the COMT content was low in the ROT + Sal and VEH groups. Therefore, this explanation is unreasonable. The second more probable explanation is that COMT activity is inhibited in the peripheral blood and tissue, and its content is upregulated as a result of negative feedback regulation. S-COMT is widely distributed in the peripheral tissues and is mainly involved in the deactivation of active and toxic catecholamines and their metabolites [36]. Reduced levels of S-COMT occurred after ROT administration, which may be related to its involvement in the metabolic process of ROT [37, 38]. Therefore, an increase in S-COMT levels in the plasma and liver may help the rats maintain a healthy state (Figure 7(a), *P* < 0.01, *P* < 0.05). In addition, there was no significant difference in the COMT content in the ST among all groups detected by ELISA (Figure 6(a)). Moreover, the decreased content of MAO-B in the ROT + *L* and ROT + Madopar® groups in the ST (Figure 7(b)) may contribute to preserving the residual DA in the brain and finally relieving PD-related symptoms.

Oxidative stress is a common pathological mechanism in PD. Many Chinese herbal compounds or herbs have been shown to have antioxidant and neuroprotective effects [39–41]. Nrf2/HO-1 is an important signaling pathway that can effectively remove ROS, mitigate the accumulation of *α*-syn, and protect neurons [42, 43]. The expression level of HO-1 increased in the ROT + *L* and ROT + *H* groups in the ST, indicating that BSJPD may activate the HO-1 pathway to relieve ROS damage to DA neurons. However, we did not find Nrf2 upregulation in the ST and it was also not observed in the SN. Thus, these findings indicate that the beneficial action of BSJPD may be associated with HO-1 but not through the Nrf-2 pathway.

We noted poor performance in the Madopar® treatment group compared to the other groups, which may be related to its mechanism. Madopar® is used only as an alternative treatment to supplement the deficiency of DA in the ST, and it has no effect on decreasing the loss of DA neurons in the SN. In addition, Madopar® treatment may lead to gastrointestinal dysfunction and abnormal dystonia, thereby damaging the health of rats. During the first week of ROT administration, a key period of high mortality occurred in rats owing to the nonspecific toxicity of ROT. Moreover, the rats that survived the acute poisoning stage gradually showed resistance to ROT. This abnormal performance may have led to variations in the final experimental results.

## 5. Conclusion

In conclusion, many classical pathological features of PD were replicated in middle-aged rats, however, some variations persisted. No obvious synergistic effect was observed between BSJPD and Madopar®; however, there might have been a slightly enhanced effect (Figure 5(f)). Our results suggest that BSJPD has a neuroprotective effect that may be related to the decreased level of MAO-B and upregulation of HO-1 expression in the ST. In addition, our findings may provide new suggestions to further investigate the underlying mechanisms of Chinese herbal compound treatment for PD. Nevertheless, this study has some limitations. First, we did not measure the level of DA in the ST; therefore, we could not directly evaluate changes in the level of DA. Furthermore, ROT can cause oxidative stress and inflammation, damaging DA neurons. However, we did not measure inflammatory markers and oxidative stress markers in our study. Furthermore, the content and activity of the enzymes were also important indicators to evaluate the biochemical action of the enzymes. At present, we have only studied the change in MAO-B content, and we will determine the activity in our next study. Therefore, the potential mechanisms by which BSJPD ameliorates these primary pathologies remain elusive and further studies are required to provide further insights.

## Figures and Tables

**Table 1:** The herbal composition of BSJPD.

Herbs	Dose (g)
*Pueraria lobata* (Willd.) Ohwi	60
*Cinnamomum cassia* Presl	10
*Paeonia lactiflora* Pall.	10
*Rehmannia glutinosa* Libosch. (RADIX REHMANNIAE)	15
*Prunus armeniaca* L.	10
*Atractylodes macrocephala* Koidz.	30
*Citrus aurantium* L.	15
*Rehmannia glutinosa* Libosch. (RADIX REHMANNIAE PREPARATA)	15
*Acorus tatarinowii* Schott	10

**Table 2:** Acute toxic effect of rotenone in rats (0–14 day).

Group	Date of onset	No. of rats		Severe Parkinson-like symptoms	Cause of death
		Onset	Death		
VEH	None	0	0	Symptomless	None
ROT + Sal	3 day, 5 day, 7 day	3	1	Bradykinesia, rigidity, postural instability	Severe weight loss, indigestion, lost the ability to drinking or feeding, iliac passion
ROT + *L*	None	0	0	Symptomless	None
ROT + *H*	7day	1	1	Bradykinesia, rigidity, postural instability	Severe weight loss, indigestion, lost the ability to drinking or feeding, iliac passion
ROT + Madopar®	7 day, 11 day	3	1	Bradykinesia, rigidity, postural instability	Severe weight loss, indigestion, lost the ability to drinking or feeding, iliac passion
ROT + *L* + Madopar®	None	0	0	Symptomless	None

*Note*. Date of onset: the time of Parkinson-like symptoms began to appear after rotenone injection.

## Data Availability

The figures and tables supporting the results of this study are included in the article and supplementary material, and the original datasets are available from the first author or corresponding author upon request.

## Supplementary Materials

Supplementary Materials 1. Original data. 2. Immunofluorescence staining. 3. OFT-track plot. The raw data and the original full-length pictures are provided in the Supplementary Material.
