# Supplementary material for: Therapeutic and Neuroprotective Effects of Bushen Jianpi Decoction on a Rotenone-Induced Rat Model of Parkinson's Disease
Source: Evid Based Complement Alternat Med. 2022 Nov 18;2022:9191284. doi: 10.1155/2022/9191284 (PMC9699733; doi:10.1155/2022/9191284)
Supplement: Supplementary Materials — 1. Original data. 2. Immunofluorescence staining. 3. OFT-track plot. The raw data and the original full-length pictures are provided in the Supplementary Material. [file 9191284.f1.zip › Original data.docx]

**1. ELISA-COMT**

1.1 LIVER-Data


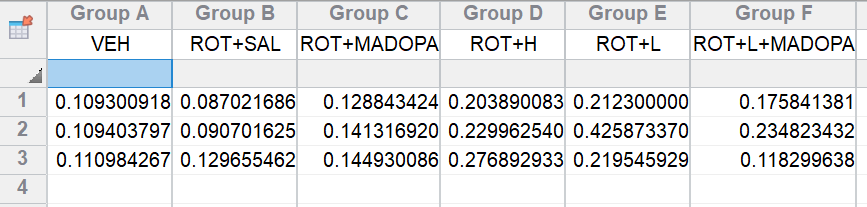


1.2 PLASMA-Data


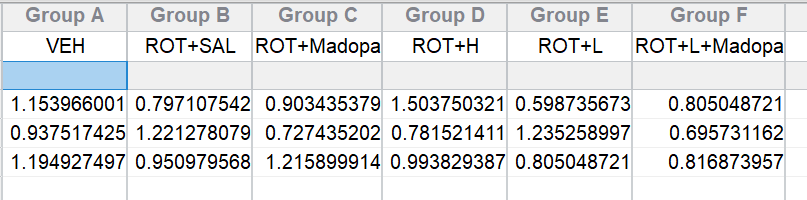


1.3 STRIATUM-Data


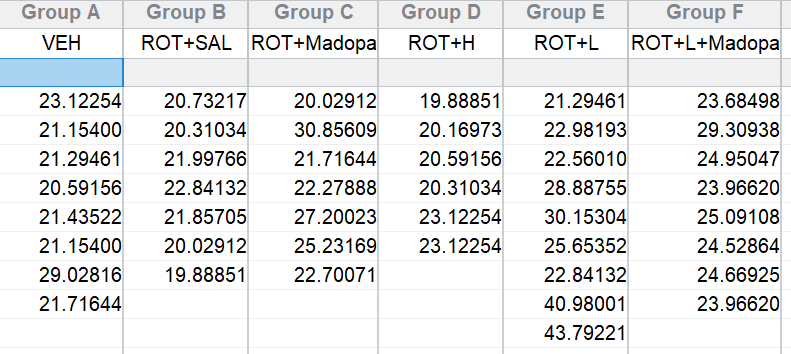


**2. ELISA-MAO-B**

2.1 LIVER-Mean


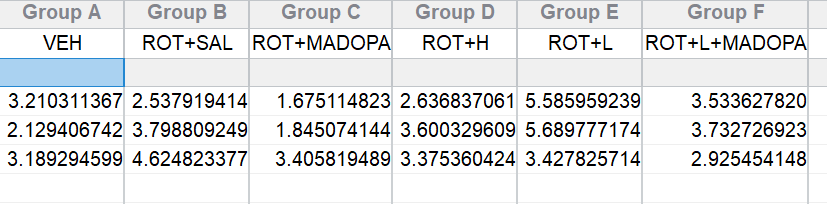


2.2 PLASMA -Mean


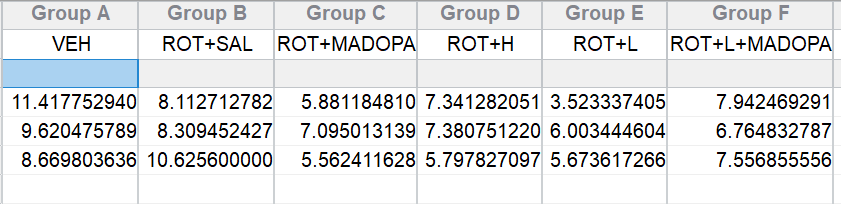


2.3 STRIATUM -Mean


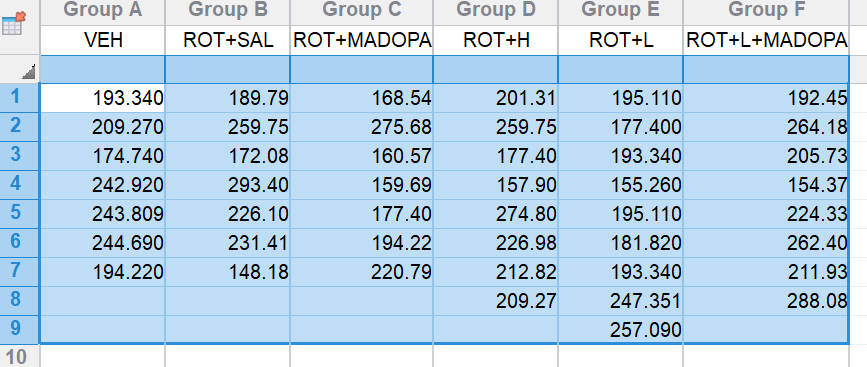


**3. HO-1-Western-blot**

3.1 SN-HO-1


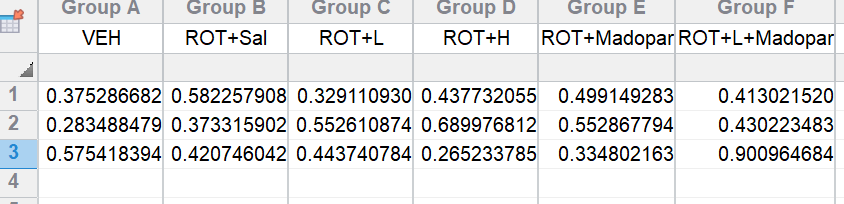


3.2 SN-HO-1-WB


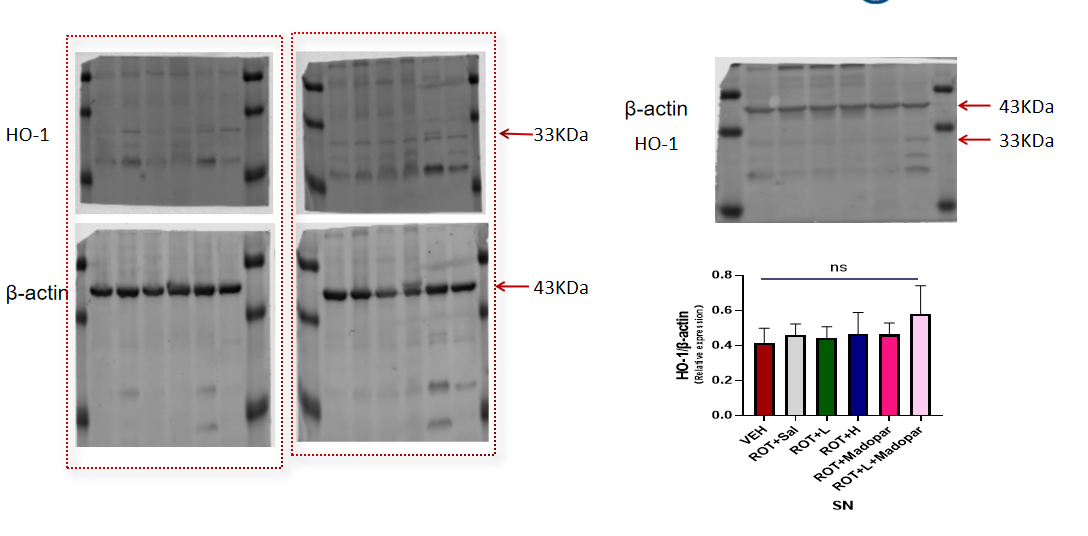


3.3 ST-HO-1-WB


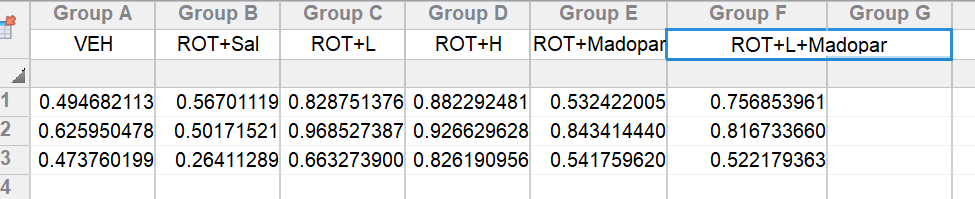

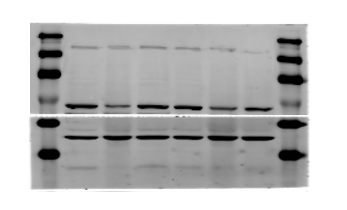

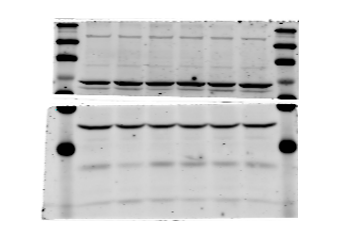

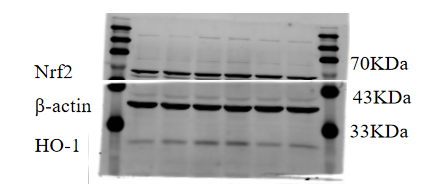


3.4.2 ST-HO-1-2

3.4.3 ST-HO-1-3

3.4.1 ST-HO-1-1

**4. Open Field Test**

4.1 Distance-mean


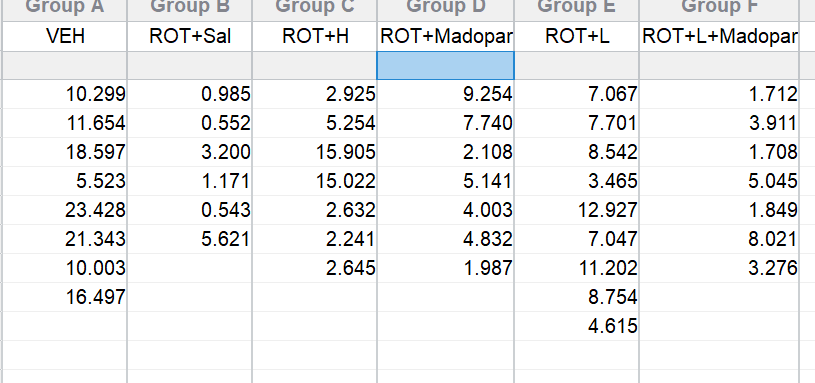


4.2 Speed-mean


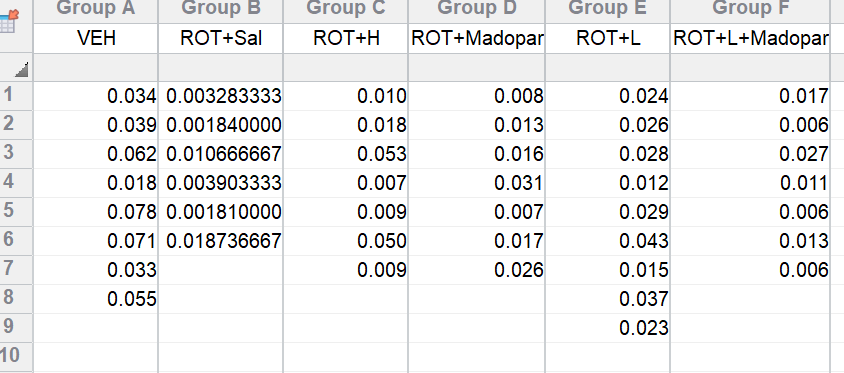


**5. Rotaroad**

Mean


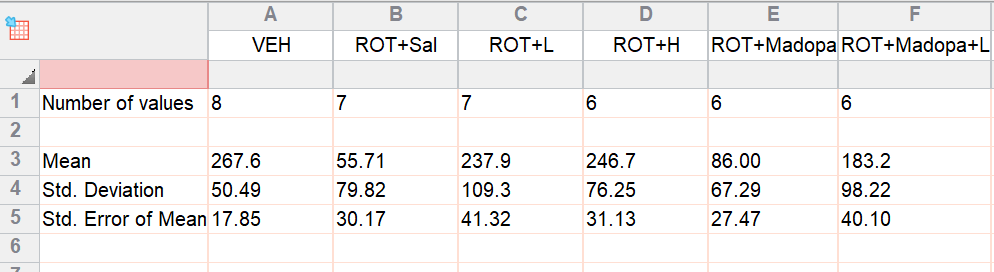


**6. SN-COMT-Western blot**

6.1 Mean


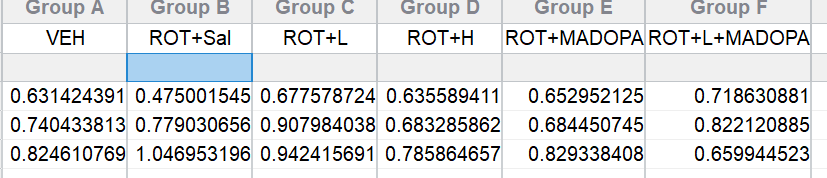


6.2 SN-COMT-1-β-actin


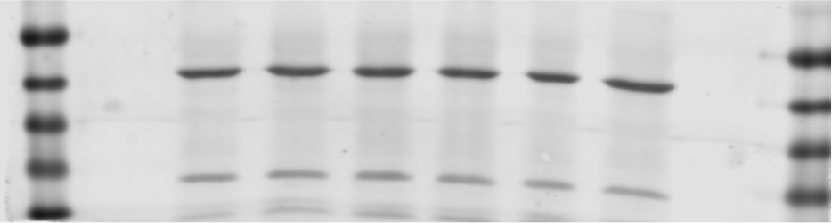


6.3 SN-COMT-2


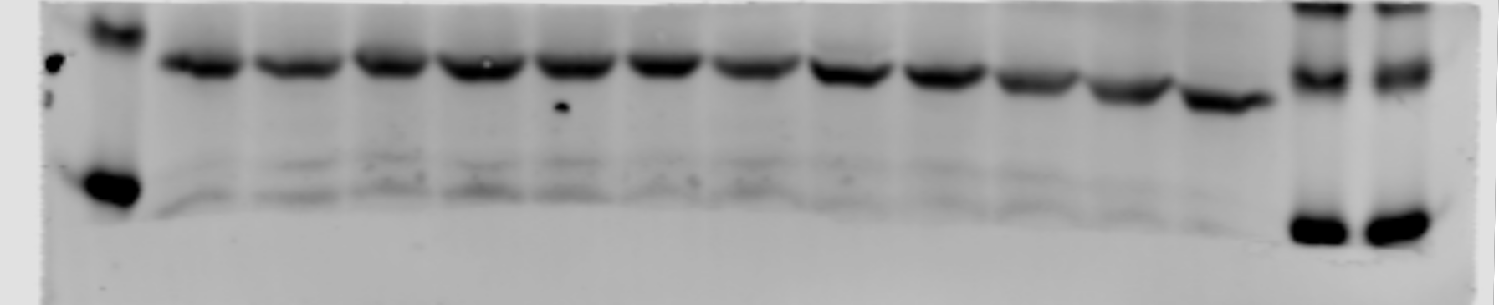


6.4 SN-COMT-2-β-ctin


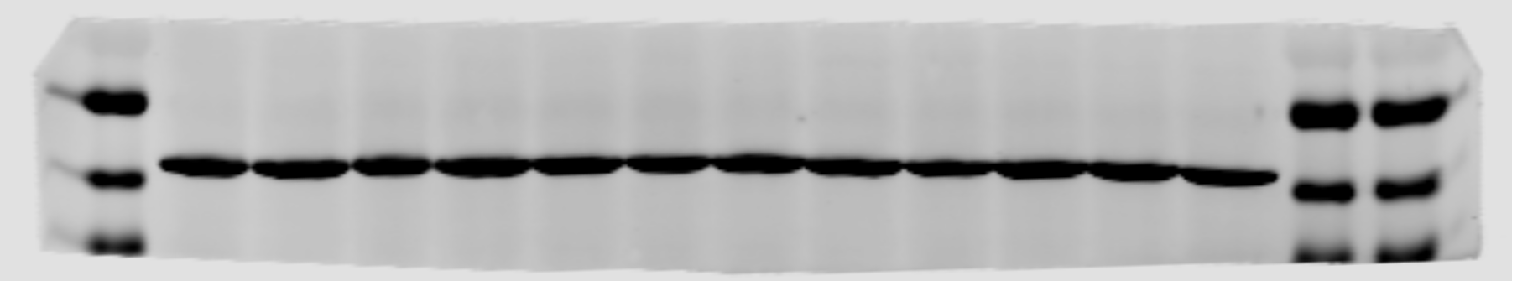


**7. SN-MAO-B-Western blot**

7.1 Mean


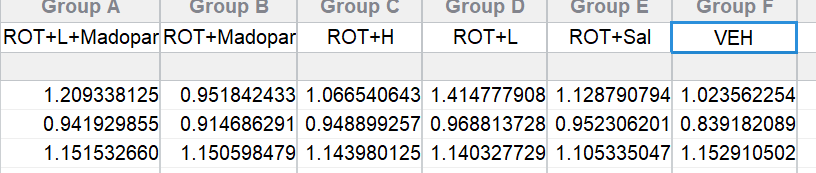


7.2 SN-MAO-B-1


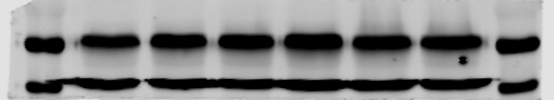


7.3 SN-MAO-B-2


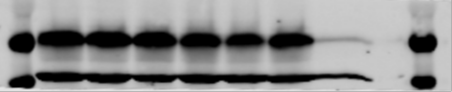


7.4 SN-MAO-B-3


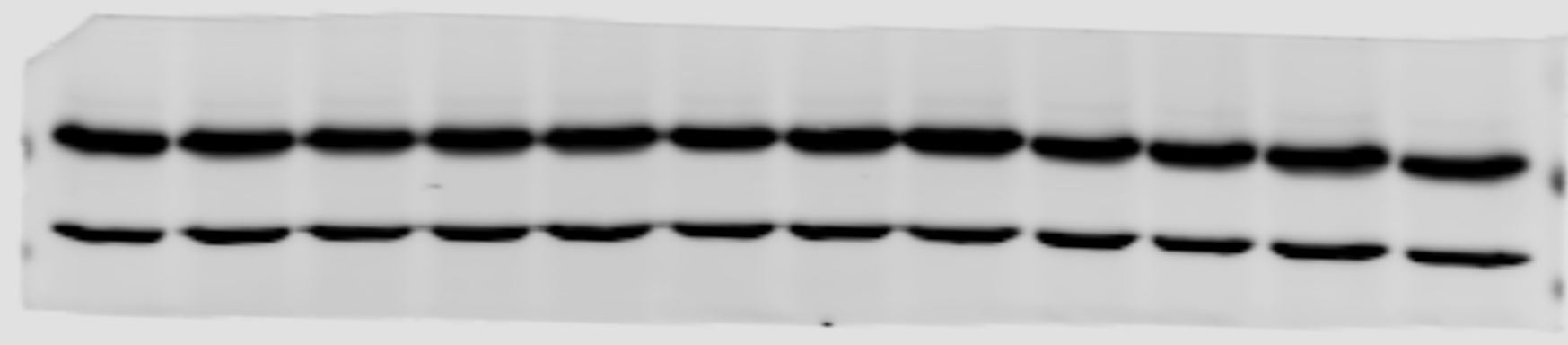


**8. STRIATUM-COMT-Western blot**

8.1 MB-COMT-Mean


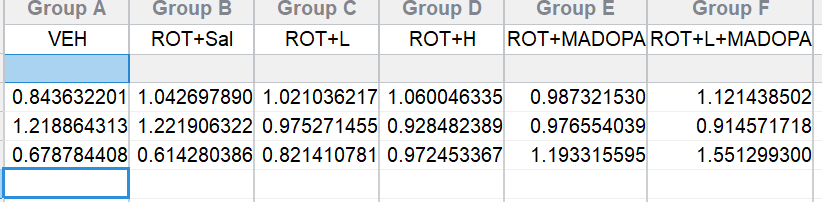


8.2 S-COMT-Mean


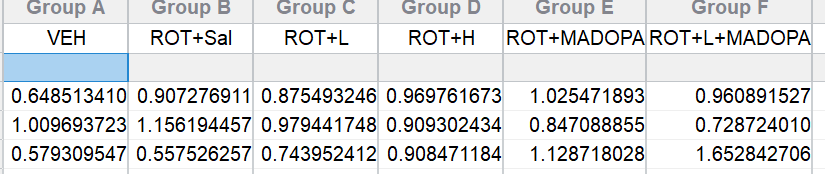


8.3 STRIATUM-COMT


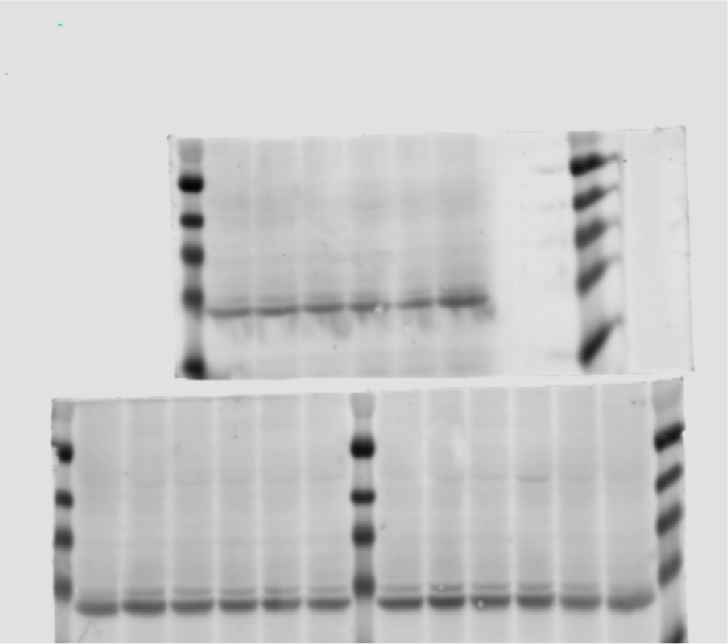


8.4 STRIATUM-β-actin (1)


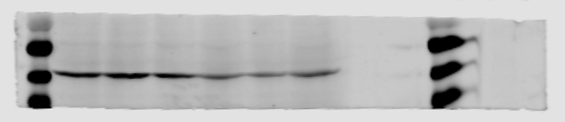


8.5 STRIATUM-β-actin (2)


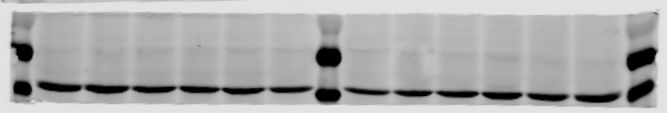


**9. STRIATUM-DDC-Western blot**

9.1 Mean


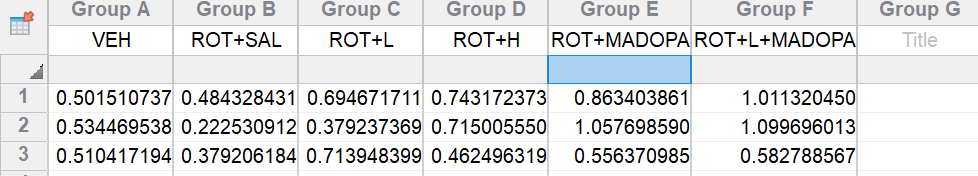


9.2 STRIATUM-DDC-1


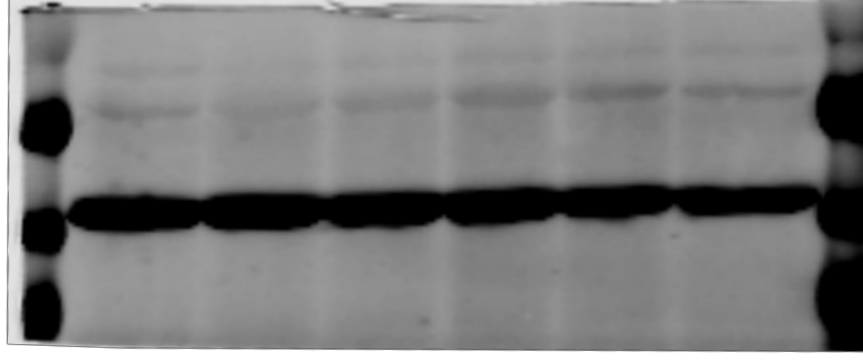


9.3 STRIATUM-DDC-2-3


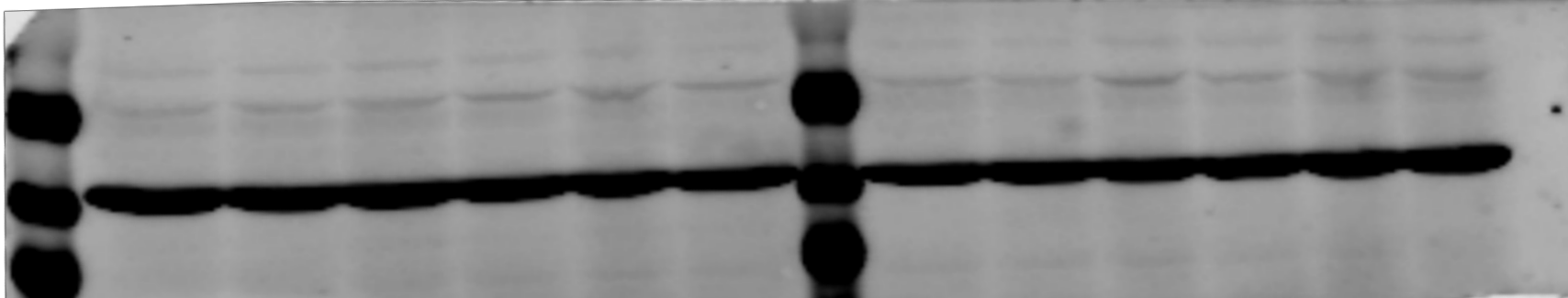


**10. STRIATUM-MAO-B-Western blot**

10.1 Mean


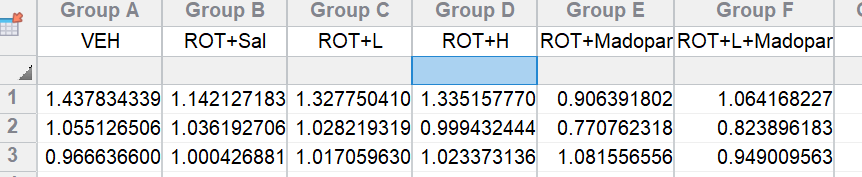


10.2 STRIATUM-MAO-B-1


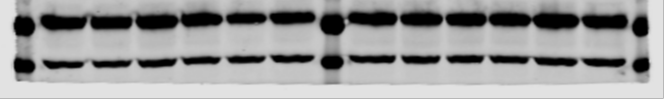


10.3 STRIATUM-MAO-B-2


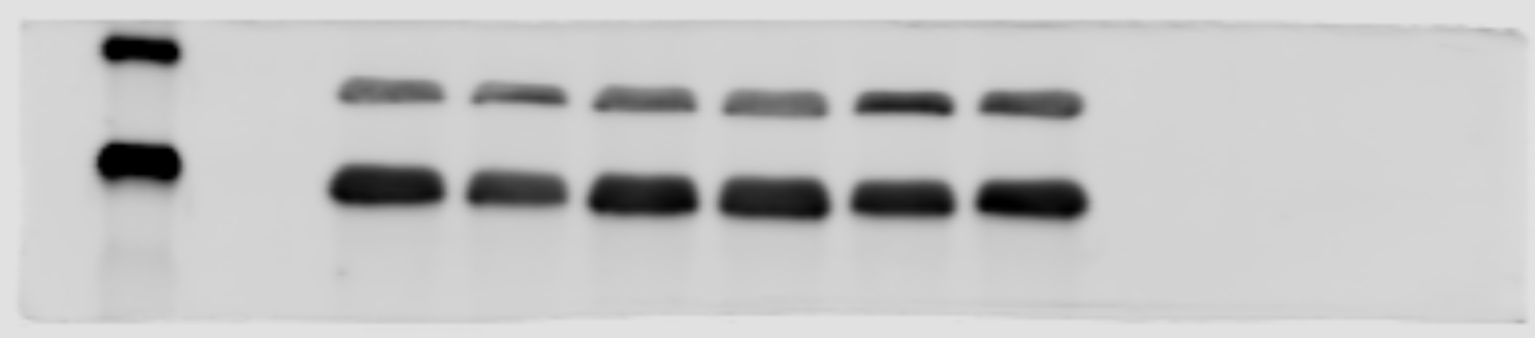


**11. TH and alpha-synuclein-Western blot**

11.1 SN-TH-Mean


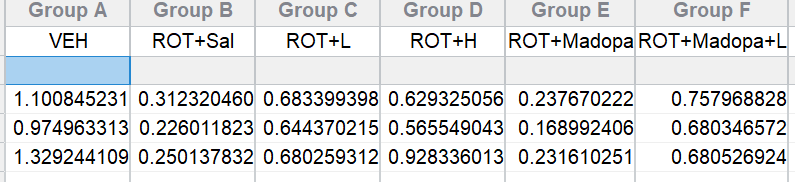


11.2 SN-α-syn-Mean


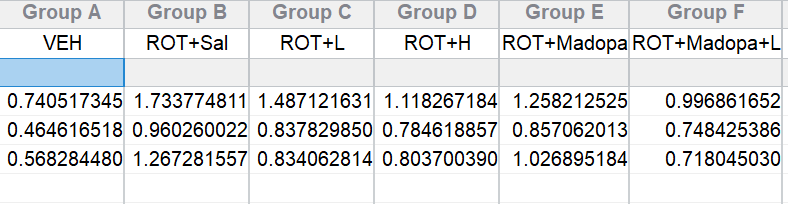


11.3 STRIATUM-TH-Mean


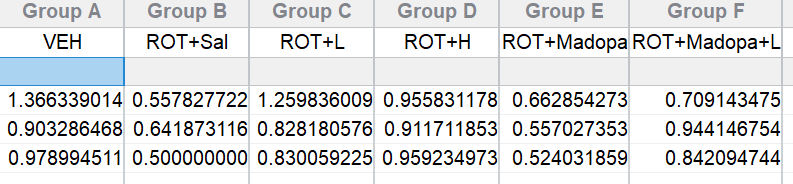


11.4 STRIATUM-α-syn-Mean


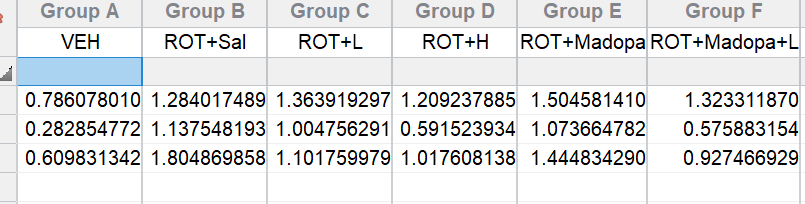


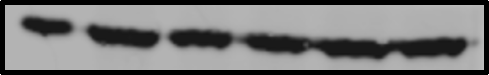


SN1-a-syn


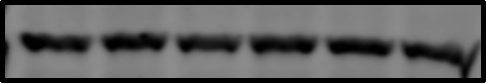

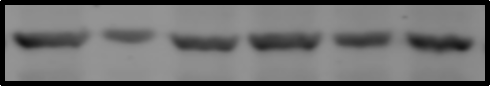

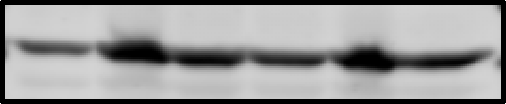

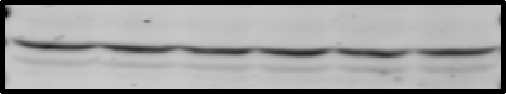

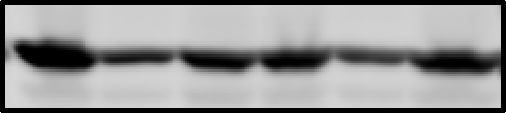

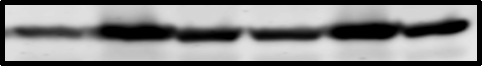

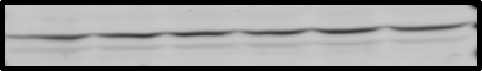

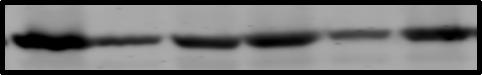


SN2-a-syn

SN1-TH

SN1-b-actin

SN2-b-actin

SN2-TH

SN3-a-syn

SN3-b-actin

SN3-TH


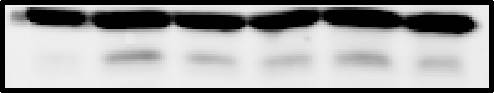

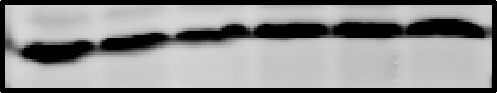

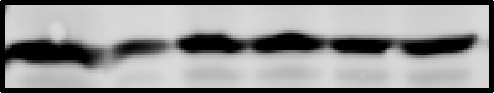

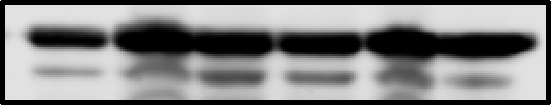

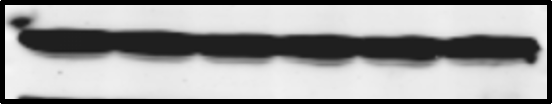

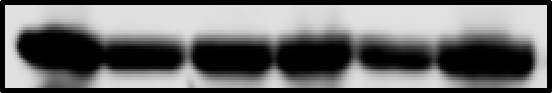

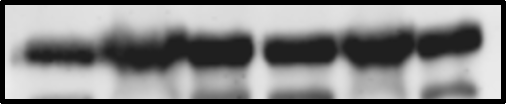

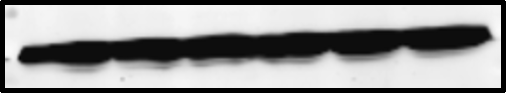

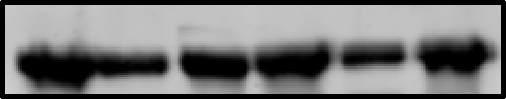


STRIATUM1-a-syn

STRIATUM1-b-actin

STRIATUM1-TH

STRIATUM2-a-syn

STRIATUM3-TH

STRIATUM3-b-actin

STRIATUM2-b-actin

STRIATUM2-TH

STRIATUM3-a-syn
